# Developing and deploying an efficient genotyping workflow for accelerating maize improvement in developing countries

**DOI:** 10.12688/gatesopenres.13338.2

**Published:** 2022-06-10

**Authors:** Queen Offornedo, Abebe Menkir, Deborah Babalola, Melaku Gedil

**Affiliations:** 1 Bioscience Center and Maize Improvement Program, International Institute of Tropical Agriculture (IITA) Headquarters, Ibadan, Oyo State, 200001, Nigeria

**Keywords:** Molecular breeding, KASP, Genotyping workflow, Marker-assisted selection, Quality Control, National Agricultural Research Systems (NARS), Developing countries

## Abstract

**Background: **Molecular breeding is an essential tool for accelerating genetic gain in crop improvement, towards meeting the need to feed an ever-growing world population. Establishing low-cost, flexible genotyping platforms in small, public and regional laboratories can stimulate the application of molecular breeding in developing countries. These laboratories can serve plant breeding projects requiring low- to medium-density markers for marker-assisted selection (MAS) and quality control (QC) activities.

**Methods: **We performed two QC and MAS experiments consisting of 637 maize lines, using an optimised genotyping workflow involving an in-house competitive allele-specific PCR (KASP) genotyping system with an optimised sample collection, preparation, and DNA extraction and quantitation process. A smaller volume of leaf-disc size plant samples was collected directly in 96-well plates for DNA extraction, using a slightly modified CTAB-based DArT DNA extraction protocol. DNA quality and quantity analyses were performed using a microplate reader, and the KASP genotyping and data analysis was performed in our laboratory.

**Results: **Applying the optimized genotyping workflow expedited the QC and MAS experiments from over five weeks (when outsourcing) to two weeks and eliminated the shipping cost. Using a set of 28 KASP single nucleotide polymorphisms (SNPs) validated for maize, the QC experiment revealed the genetic identity of four maize varieties taken from five seed sources. Another set of 10 KASP SNPs was sufficient in verifying the parentage of 390 F _1_ lines. The KASP-based MAS was successfully applied to a maize pro-vitamin A (PVA) breeding program and for introgressing the aflatoxin resistance gene into elite tropical maize lines.

**Conclusion: **This improved workflow has helped accelerate maize improvement activities of IITA's Maize Improvement Program and facilitated DNA fingerprinting for tracking improved crop varieties. National Agricultural Research Systems (NARS) in developing countries can adopt this workflow to fast-track molecular marker-based genotyping for crop improvement.

## Introduction

Agriculture is the mainstay of millions of low-income households in Sub-Saharan Africa ( SSA). However, productivity is way below the yield potential of significant crops due to several interacting factors contributing to the yield reduction. The paucity of nutritionally improved resilient crop varieties is a crucial constraint. This constraint can be mitigated by the rapid development of cultivars adapted to specific agroecology zones ^1^. The current yield gain trend in major food crops has shown that relying on conventional breeding alone is insufficient to meet the food needs of an estimated nine billion people in 2025 ^2^. There is a need to accelerate genetic gain by deploying new breeding strategies ^3, 4^. This need has led to the scientific community's massive investment in developing genomic resources and support systems, to provide valuable tools to accelerate breeding processes ^5^.

Various bottlenecks have hindered the substantial impact of molecular breeding for crop improvement, particularly in developing countries ^6, 7^. The major limiting factors are a lack of infrastructure and capacity for genomics resources and poor information flow, resulting in reduced access to operational and decision support tools ^8^. Private companies in developed countries usually own the proprietary rights to many emerging genomics resources and systems, making it difficult for public research sectors, non-profit research institutes, and small laboratories in developing countries to have direct access. These challenges are being curbed by various international initiatives such as the Excellence in Breeding (EiB) platform, which coordinates its activities with the Genomic and Open-source Breeding Informatics Initiative (GOBii), and High Through-Put Genotyping (HTPG). In addition, the Integrated Breeding Platform (IBP)-hosted Generation Challenge Program (GCP) and the Breeding Management System (BMS) ^9^ target the development and adoption of molecular breeding in developing countries. These and other consultative group-hosted initiatives and platforms galvanise worldwide partners drawn from public, private, and governmental institutions towards the common goal of increasing agricultural productivity through efficient tools, technologies, and data management systems ^6^.

Despite the availability of many low-cost genotyping platforms and resources, it is not easy to meet the genotyping needs of many users who work on different crops, different locations, and often fewer samples due to cost implications ^7, 8^. The current available genotyping platforms have a minimum sample size requirement. For instance, the EiB facilitated genotyping at Intertek offers reduced cost if the user orders genotyping of 1536 samples; fewer samples are acceptable but the price increases. However, breeders often want to fingerprint a few dozen lines urgently for identity or parentage analysis for example. In such cases, sending less than the minimum number of samples is not only more priced per datapoint but entails shipping cost and a turn-around time of 2–3 weeks. Using other markers, such as SSR, is more expensive and cumbersome. The use of genotyping systems such as KASP in-house alleviates all these issues. Also, the issue of inefficient courier services in this part of the world, which often results in reduced or damaged perishable specimens, can be circumvented if a reasonably affordable system is available locally. More so, we re-purposed standard laboratory instruments for the genotyping workflow. For instance, the qPCR machine, which is mostly used for expression analysis, was adapted to KASP genotyping with the installation of appropriate software for SNP calling. Likewise, the Fluostar plate reader was used for plate-level DNA quantification in lieu of single sample analysis by Spectrophotometer.

For these reasons it is imperative to devise a sustainable strategy for routine, cost-effective, and easily accessible genotyping services to complement these international outsourcing initiatives by providing in-house or local (regional) genotyping platforms, where possible, to accelerate the genotyping workflow. One such regional initiative in Africa is the Integrated Genotyping Support Services (IGSS) genotyping facility at Biosciences eastern and central Africa/International Livestock Research Institute ( BeCA/ILRI), Kenya. This strategy will allow breeders to outsource to a regional genotyping service provider or set up a core facility in-house.

One factor that influences breeders' choice of genotyping platform is the level of throughput. Other factors considered are the data turnaround time, ease of data analysis (available informatics), reproducibility, flexibility, and cost per datapoint or cost per sample ^10, 11^. For high and ultra-high throughput markers, breeders outsource to array- and sequenced-based genotyping service providers. These platforms are suitable for discovery applications and approaches requiring hundreds to thousands of samples to be genotyped with tens to thousands of markers, such as genome-wide association studies (GWAS), gene mapping, and large-scale genomic selection ^10, 12^ They are also suitable for genotyping a few samples with many markers (multiplexing), such as genetic diversity analysis or background selection. While multiplex platforms provide higher throughput with lower reagent consumption, it limits scientists to using a multiplexed set of several thousand single nucleotide polymorphisms (SNPs) per assay ^13^. They are also demanding in informatics resources and presently produce datasets with a significant percentage of missing data ^13^. The high cost per sample and the initial assay development time of highly multiplexed platforms can be problematic for crop improvement applications, usually requiring low- to medium-density markers ^11^. For these low- to mid-density genotyping approaches, a uniplex SNP genotyping platform is appropriate ^14^.

Uniplex genotyping assays are low-throughput genotyping systems that are ideally flexible regarding assay design, ease of running, and cost-effectiveness ^15^. These systems provide plant breeders with the flexibility to mix and match different SNPs for a given sample set. They allow breeders to use a smaller subset of informative SNPs such as functional SNPs and trait-specific haplotypes, thereby eliminating the generation of unintended datapoints when using fixed-array SNPs. Even though a range of uniplex SNP genotyping assays exists, the most competitive uniplex systems that have been successfully applied in crop improvement research are TaqMan ^16– 19^, competitive allele-specific PCR (KASP) ^11, 20^, Amplifuor ^21^, and rhAmP ^22^ assays. These uniplex genotyping systems vary in reaction chemistry, detection method, and reaction format. Uniplex systems can either be outsourced or installed in-house.

Here we utilised the KASP assay, as it is one of the most used assays among plant breeders and biologists ^15, 19^. KASP is an endpoint PCR-based SNP genotyping method from KBiosciences, now LGC Biosearch Technologies, UK. KASP uses fluorescently-labelled allele-specific primers for the bi-allelic discrimination of SNPs and insertion-deletion mutations (INDELs) ^23^. KASP was developed to reduce cost, mainly from probe design, and improve genotyping efficiency, becoming a preferred alternative to TaqMan ^11, 24^. The KASP genotyping system has been successfully applied in crops such as maize ^11, 15^, wheat ^10, 20, 25^, rice ^26^, soybean ^27^, peanut ^28^, amongst others. KASP has developed into a global benchmark technology for genotyping crop plants ^11, 23, 28– 30^ following the validation of KASP markers across crops of global importance (such as maize - 1250 markers, wheat - 1864 markers, and rice - 2015 markers) by the Generation Challenge Program of the Integrated Breeding Platform ^9^. The International Maize and Wheat Improvement Center (CIMMYT) has successfully utilised the 1,250 maize KASP markers for various genetic applications, including quantitative trait loci (QTL) mapping, marker-assisted recurrent selection (MARS), allele mining, and QC analysis ^11^. The Maize Improvement Program of the International Institute of Tropical Agriculture (IITA) has generated over 2,000 datapoints using KASP in-house for different genotype analyses, including QC and MAS.

However, some bottlenecks in the genotyping workflow slow down the genotyping process, delaying crop improvement: (1) method of sample collection and processing, (2) level of DNA extraction and quantitation, and (3) DNA-based genotyping ^8^. Gedil and Menkir (2019) provided a thorough review of the Maize Improvement Program's (MIP) molecular marker-based crop improvement activities. However, reports of research accelerating the entire genotyping process by minimizing these bottlenecks and providing a cost-effective genotyping workflow suitable for small scale breeders and laboratories in developing countries are lacking. This work aimed to show how deploying an in-house genotyping platform alongside optimised molecular techniques can accelerate maize improvement activity for a quick result turnaround. It provides a workflow that less sophisticated breeding laboratories can also adopt for efficient crop improvement in developing countries.

## Methods

### Plant materials

The overall genotyping workflow was applied in some experiments representative of the genotyping activities common in small to medium breeding programs. Table 1 below describes the plant materials used in each experiment. The genetic identity experiment was performed using four well-adopted maize varieties originating from IITA but regenerated at four locations. For the hybrid verification experiment, 60 maize F _1_ progenies originating from five bi-parental crosses were used. Lines KS23-3, KS23-5, and KS23-6 are resistant to maize lethal necrosis (MLN) disease, while IITATZI1653 and IITATZI1667 are IITA-adopted elite maize lines with high PVA content. Another 330 F _1_ plants originating from four sets of bi-parental crosses involving *Striga*-susceptible (TZdEEI 102, TZdEEI 99, TZdEEI 4, and TZdEEI 13) and *Striga*-resistant (TZEEI 29, and TZEEI 79) parents were also screened to identify true hybrids. A total of 70 PVA-QPM enriched maize inbred lines were genotyped to select lines harbouring the favourable allele for the *crtRB1* gene associated with PVA content in maize. In the fourth breeding cycle of the maize enrichment project using marker-assisted backcrossing to introgress resistance to aflatoxin accumulation in elite tropical maize lines, we genotyped a total of 159 BC _1_S _2 _maize lines. We applied a 15% selection intensity to identify lines harbouring the favourable alleles of the QTLs associated with resistance to aflatoxin accumulation. These plants were raised in maize fields at IITA Ibadan, Nigeria.

**Table 1.** Plant materials used for the experimentation of the optimized genotyping workflow.

S/N	Experiments	Parental maize lines: traits	Bi-parental crosses	Sample development
1	**Genetic identity**	• **SAMMAZ 15 (IWDC2SynF2)**: Medium-maturing, good seed quality, high yield potential, tolerance to Striga hermonthica. (Y-6.9t/ha) • **SAMMAZ 16 (TZLComp1SynW-1):** Late maturing, good seed quality, high yield, resistance to Striga hermonthica. (6.4t/ha) • **SAMMAZ 27 (EV99DT-W-STR):** Drought tolerant and Striga resistant. (5.5t/ha) • **SAMMAZ 39 (PVA SYN8)**: Intermediate-level pro-vitamin A content (6.4µg/g), high yield potential. (6.8t/ha)		Performed using four well-adopted maize varieties originating from IITA but regenerated at four locations. Maize seedlings were raised in pots for about two weeks until they reached the three-four-leaf stage in a screen house at the Bioscience Center of IITA Ibadan, Nigeria.
2	**Hybrid verification:** Set a: 60 F _1_ Set b: 330 F _1_	• **KS23-3, KS23-5, and KS23-6**: resistant to maize lethal necrosis (MLN) disease • **IITATZI1653 and IITATZI1667:** high PVA content • **TZdEEI 102, TZdEEI 99, TZdEEI 4, and TZdEEI 13**: Striga susceptible • **TZEEI 29, and TZEEI 79**: Striga resistant	**Set 1a**: KS23-3 x IITATZI1653; **Set 2a**: KS23-5 x IITATZI1653; **Set 3a**: KS23-6 x IITATZI1653; **Set 4a**: KS23-3 x IITATZI1667; **Set 5**: KS23-5 x IITATZI1667; **Set 1b**: TZEEI 29 x TZdEEI 99; **Set 2b**: TZdEEI 4 x TZEEI 79; **Set 3b**: TZEEI 79 x TZdEEI 13; **Set 4b**: TZdEEI 102 x TZEEI 29	Seedlings for the F1 plants were raised in a maize field at IITA Ibadan, Nigeria.
3	**Marker-assisted** ** selection**	• **70 PVA-QPM** enriched maize inbred lines were genotyped to select lines harbouring the favourable allele for the crtRB1 gene associated with PVA content in maize • **159 BC1S2** maize lines in the fourth breeding cycle of the maize enrichment project using marker-assisted backcrossing to introgress resistance to aflatoxin accumulation in elite tropical maize lines		Seedlings of the inbred maize lines were raised in a maize field at IITA Ibadan, Nigeria. Ten plant stands per row were planted for each inbred, and leaf tissues were collected from each row for DNA extraction by bulking leaves from all ten plant stands per row. For the aflatoxin population, we applied a 15% selection intensity to identify lines harbouring the favourable alleles of the QTLs associated with resistance to aflatoxin accumulation. These plants were raised in a maize field at IITA Ibadan, Nigeria

**Legend: PVA = Pro-vitamin A; QTL = Quantitative trait loci. Source of plant materials**: Maize Improvement Program, International Institute of Tropical Agriculture (IITA) Headquarters, Ibadan, Nigeria.

### Sample collection and preparation, and DNA extraction and quantitation

A total of 16 to 20 leaf discs were collected from young leaves of each tagged plant, directly into Corning 96-well Polypropylene 1.2 ml cluster tubes with strip caps (Merck, Germany) using Haris Uni-core 4.0 mm puncher and cutting mat (Merck, Germany). Two 4.0 mm stainless steel grinding balls (SPEX SamplePrep) were placed in each tube. Plant tissues were preserved on ice for transport from the field to the laboratory. They were stored in a -80°C freezer before lyophilising for 48 hours using FreeZone Freeze Dryer (Labconco) following the manufacturer's manual. Lyophilised leaf tissues were ground into powder by shaking at 1,500 strokes per minute for 1.5 min using an automated high-throughput tissue homogeniser, Geno/Grinder 2010 (SPEX SamplePrep).

Genomic DNA was extracted from ground leaf tissues using a cetyltrimethylammonium bromide (CTAB)-based DNA extraction method as described by Diversity Array Technology (DArT) ^31^ with minor modifications ( Table 2). Dry leaf tissues were used instead of fresh ones; we included a 30-minute incubation period during the alcohol precipitation step; the DNA pellet was resuspended in a nuclease-free water and RNaseA solution. The DNA quality and quantity were determined by spectrophotometry using the FLUOstar Omega Microplate Reader (BMG LABTECH) following the manufacturer's manual.

**Table 2.** DArT DNA extraction protocol with minor modification.

The chemicals and reagents used were as outlined in the Diversity Array Technology (DArT) Plant DNA extraction protocol (Accessed on June 2, 2020). **Extraction procedure:** 1. Aliquot freshly prepared, well-mixed "fresh buffer solution" and preheat in a 65°C water bath. 2. Grind sample leaf discs in 1.2 ml cluster tubes using a Geno/Grinder 2010 (Spex Sample Prep) to a fine powder 3. Add 500 μl buffer solution to dissolve the powder completely 4. Incubate at 65°C for 1 hr, with gentle shaking 5. Cool down for 5 min and add 500 μl of chloroform: isoamyl alcohol (24:1) mixture 6. Mix well by gentle inversion for 30 min, and spin for 20 min, at 10,000 x g, at room temperature 7. Transfer about 400 μl of the water phase to a fresh 1.2 ml tube, add the same volume of ice-cold isopropanol and invert the tube approximately ten times, nucleic acids should become visible 8. Incubate for 30 min at -20 °C, and spin for 30 min, at 10,000 x g, at room temperature 9. Discard supernatant, and wash pellet with 400 μl 70 % EtOH 10. Discard EtOH, dry pellet and dissolve in 100 µl of nuclease-free water-RNAseA solution in a 90:10 ratio.

### KASP genotyping and data analysis

The isolated genomic DNA was diluted to a working concentration of 30 ng/µl and used as template DNA for the KASP genotyping reaction. A total of 28 KASP SNPs were used to determine the selected maize varieties' genetic identity, while 10 KASP SNPs were used to verify true hybrids among the F _1_ maize lines. The SNPs ( Table 3) were taken from a maize QC SNP panel ^9^ recommended by CIMMYT ^7, 32^ and chosen for their high polymorphic information content (PIC) and uniform maize genome coverage. Trait-specific KASP markers ( Table 4) were used to screen BC _1_S _2_ lines carrying the favourable allele for resistance to aflatoxin accumulation and identify inbred lines with high PVA content. The KASP reaction was performed in 96- and 384-well plates. For the 96-well plate, a total reaction volume of 10 µl consisting of 5 µl template DNA and 5 µl of the prepared genotyping mix (2×KASP master mix and primer mix) was used. In contrast, for the 384-well plate, a total reaction volume of 5 µl consisting of 2.5 µl template DNA and 2.5 µl of the prepared genotyping mix was used. All reaction was performed following the KASP manual (accessed on June 24, 2020). The KASP assay and master mix were purchased from LGC Biosearch Technologies (LGC Group). The amplification reaction was run in-house (Bioscience Centre of IITA Ibadan, Nigeria) using the LightCycler 480 II PCR System (Roche Life Sciences, Germany) and GeneAmp PCR System 9700 (Applied Biosystems, USA). The description of the parameters for the LC480 II qPCR machine is outlined in the LC480 operator’s manual. To perform the KASP genotyping experiment on the LC480 II machine, we used the Endpoint Genotyping Analysis module within the LightCycler software, adjusting the parameters as outlined in the KASP genotyping protocol provided by LGC Biosearch Technologies. The Endpoint genotyping analysis module is based on the use of dual hydrolysis probes, which are designed for wild-type and mutant target DNA and are labelled with different dyes (FAM and HEX). However, when using a non-qPCR machine (such as the GeneAmp PCR System 9700) for amplification, a third colour probe (ROX) normalizes the fluorescence measurement. The LightCycler software within the LC480 II machine determines the sample genotypes automatically by measuring the intensity distribution of the two probes after a PCR amplification step. The relative dye intensities are then visualized in a scatter (cluster) plot that discriminates them as wild-type, heterozygous mutant, or homozygous mutant samples. The LightCycler software automatically groups similar samples and assigns genotypes based on the intensity distribution of the two dyes. The KASP amplification conditions included one cycle of KASP unique Taq activation at 94°C for 15 min, followed by 36 cycles of denaturation at 94°C for 20 s, and annealing and elongation at 60°C (dropping 0.6°C per cycle) for 1 min. Endpoint detection of the fluorescence signal was acquired for 1 min at 30°C when using the LightCycler 480 II real time-PCR System or read using the FLUOstar Omega Microplate reader (BMG Labtech, SA) when using the GeneAmp PCR System 9700. For fluorescence detection, the filter combination for the Excitation and Emission wavelength of both dyes was set at 465 – 533 (FAM) and 523 – 568 (HEX), respectively, when using LC480 II, and 485 - 520 (FAM), 544 - 590 (HEX) and 584 - 620 (ROX) when using FLUOstar Omega Microplate reader. The genotype calls were exported from the LightCycler software as fluorescent intensities of each sample in ".txt" file format and imported for analysis in the KlusterCaller analysis software (LGC Biosearch Technologies). The KlusterCaller software adjusted the cluster plot axes to enable the proper calling of genotypes. The genotype calls were grouped as homozygous for allele X (allele reported by FAM, X-axis), homozygous for allele Y (allele reported by HEX, Y-axis), heterozygous (alleles reported by FAM and HEX, between X- and Y-axis), or uncallable. The result from the KlusterCaller was exported in two file formats (".csv" and ".txt"). The ".csv" file was imported into the SNPviewer2 version 4.0.0 software (LGC Biosearch Technologies), where the cluster plot image was viewed and downloaded for publication. The genotype calls in the ".txt" file were used to calculate the genetic distance using the PowerMaker 3.25 statistical software ^33^.

**Table 3.** List of KASP single nucleotide polymorphisms (SNPs) used in the QC experiments.

SNP ID	Linkage group	Position (cM)	Allele X	Allele Y	Trait category	Analysis	Dataset
ae1_7	5	79	A	G	QC	GID & HV	GCP/IBP-Maize
PHM15331_16	10	28	A	G	QC	GID	GCP/IBP-Maize
PHM2438_28	4	12	A	G	QC	GID	GCP/IBP-Maize
PHM2770_19	10	36	A	C	QC	GID	GCP/IBP-Maize
PHM3466_69	6	108	A	G	QC	GID	GCP/IBP-Maize
PHM5181_10	9	26	C	T	QC	GID & HV	GCP/IBP-Maize
PHM5502_31	3	58	A	G	QC	GID & HV	GCP/IBP-Maize
PZA00413_20	3	60	A	C	QC	GID & HV	GCP/IBP-Maize
PZA00726_10	4	55	A	C	QC	GID	GCP/IBP-Maize
PZA01216_1	1	116	A	G	QC	GID & HV	GCP/IBP-Maize
PZA01456_2	10	61	A	G	QC	GID	GCP/IBP-Maize
PZA01477_3	4	81	C	T	QC	GID	GCP/IBP-Maize
PZA01533_2	7	112	A	G	QC	GID	GCP/IBP-Maize
PZA01885_2	2	115	A	G	QC	GID & HV	GCP/IBP-Maize
PZA01919_2	10	44	C	G	QC	GID & HV	GCP/IBP-Maize
PZA02090_1	3	15	A	T	QC	GID & HV	GCP/IBP-Maize
PZA02164_16	5	70	A	G	QC	GID & HV	GCP/IBP-Maize
PZA02269_3	1	149	C	T	QC	GID & HV	GCP/IBP-Maize
PZA02358_1	4	31	A	G	QC	GID	GCP/IBP-Maize
PZA02378_7	2	64	A	G	QC	GID	GCP/IBP-Maize
PZA02741_1	1	91	C	T	QC	GID	GCP/IBP-Maize
PZA02746_2	8	94	G	T	QC	GID	GCP/IBP-Maize
PZA02779_1	4	108	A	G	QC	GID & HV	GCP/IBP-Maize
PZA03135_1	8	57	A	C	QC	GID & HV	GCP/IBP-Maize
PZA03363_1	7	49	A	G	QC	GID & HV	GCP/IBP-Maize
PZA03605_1	10	75	A	G	QC	GID	GCP/IBP-Maize
PZB01658_1	6	28	A	T	QC	GID & HV	GCP/IBP-Maize
sh1_12	9	18	A	G	QC	GID & HV	GCP/IBP-Maize

**LEGEND**: QC = Quality control; GID = Genetic Identity; HV = Hybrid verification; GCP/IBP = Generation Challenge Program/Integrated Breeding Platform. **Source**: Integrated Breeding Platform (Accessed June 26, 2020).

**Table 4.** List of trait-specific KASP single nucleotide polymorphisms SNPs used in the MAS experiment.

SNP ID	Chromosome No.	FAM allele	HEX allele	Trait category	analysis	Source
S1_85016181	1	C	G	Aflatoxin	MAS	CIMMYT/IITA
S3_14863214	3	G	A	Aflatoxin	MAS	CIMMYT/IITA
S3_90027035	3	A	G	Aflatoxin	MAS	CIMMYT/IITA
S3_90023939	3	T	A	Aflatoxin	MAS	CIMMYT/IITA
S3_179639685	3	C	G	Aflatoxin	MAS	CIMMYT/IITA
S3_14229695	3	T	C	Aflatoxin	MAS	CIMMYT/IITA
S5_182519023	5	A	G	Aflatoxin	MAS	CIMMYT/IITA
S5_63229636	5	C	A	Aflatoxin	MAS	CIMMYT/IITA
S5_198883041	5	T	A	Aflatoxin	MAS	CIMMYT/IITA
PHM12859_7	3	C	T	Aflatoxin	MAS	CIMMYT/IITA
PZA02792_16	5	T	C	Aflatoxin	MAS	CIMMYT/IITA
MZA4145_18	3	A	G	Aflatoxin	MAS	CIMMYT/IITA
snpZM0015	10	A	G	PVA	MAS	CIMMYT

**LEGEND**: MAS = Marker-assisted selection; PVA = Provitamin A; CIMMYT = International Maize and Wheat Improvement Center; IITA = International Institute of Tropical Agriculture.

### Source data

The list of KASP SNPs for genotyping maize was obtained freely from the Integrated Breeding Platform website.

The trait-specific KASP SNPs (Supplementary Table 1, *Underlying data*) and QC KASP SNPs (Supplementary Table 2, *Underlying data*) were purchased as KBDs (KASP-by-Design) from LGC Biosearch Technologies, UK, for use in our laboratory.

## Results

### Optimising in-house genotyping workflow

**Figure 1.**
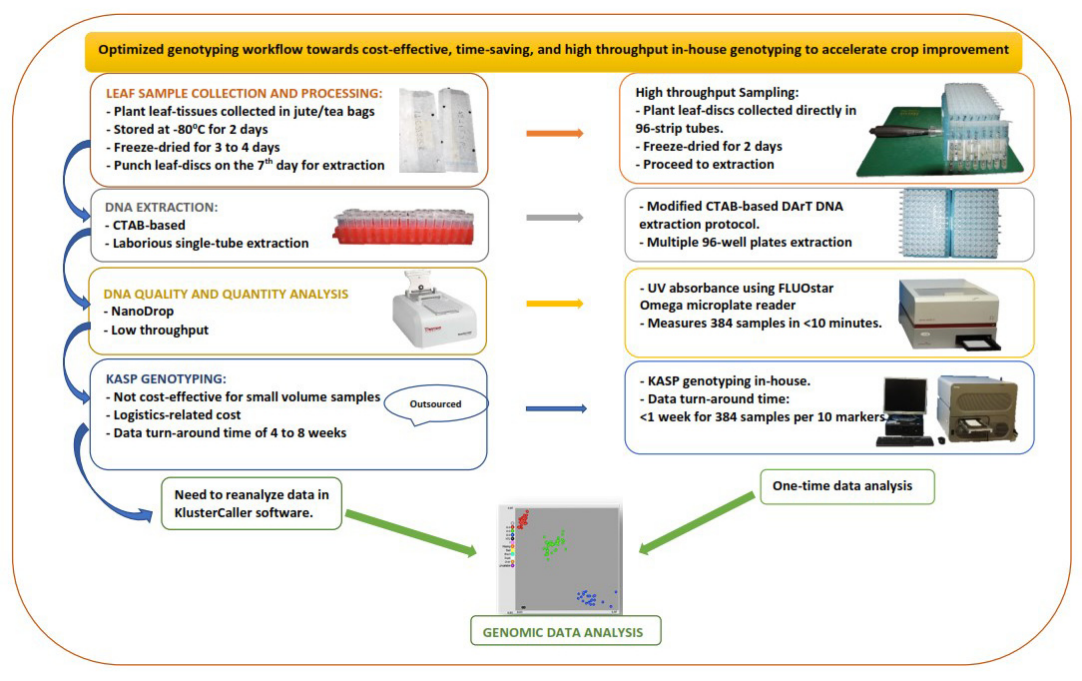
Diagram showing improvement to minimize bottlenecks in the genotyping workflow.

Our laboratory's routine sampling procedure spans seven days, from plant sampling and preparation to DNA extraction and quantitation. We present an expedited workflow ( Figure 1) that ensures a good sample tracking system. Firstly, barcoding software, barcode readers, barcode labels, and barcode printers were introduced to facilitate sample tracking and data management. Waterproof/tear-proof tags and labels designed using BarTender barcoding software (Seagull Scientific) were printed using ZT230 Printer (Zebra, USA) and attached to plants before sample collection. Plate maps created in the BarTender software were linked to the sample location on the field and in the lab storage facility. Next, young plant leaf tissues were collected by punching leaf discs directly into the 96-well 1.2 mL polypropylene cluster tubes in wet-ice cooler bags, which reduced the sampling time and the time required for freeze-drying.

The sample DNA was extracted using the DArT DNA extraction protocol, slightly modified to maximise reagent and increase throughput, by using a reduced volume of reagents optimised to extract maize DNA from a smaller amount of leaf tissue (16–20 leaf discs, 4.0 mm). We also used freeze-dried leaf tissue, which allowed grinding using an automated high-throughput tissue homogeniser, Geno/Grinder 2010, with a 384-samples grinding capacity (4 × 96-sample plates) in two minutes.

The UV absorbance protocol for the FLUOstar Omega microplate reader (BMG LABTECH) was used to measure the concentration and purity of the DNA samples. By using this method, the 637 DNA samples were quantified in less than 10 minutes. The DNA purity (A260/A280 ratio) ranged from 1.7 to 2.0, with an average concentration of 985 ng/µl.

Following the optimized workflow, the total time from sampling and processing to DNA extraction and quantitation of the 637 leaf samples was reduced from seven to five days.

In order to optimise and use the KASP system in-house, KASP assays and allele-calling software (KlusterCaller) were purchased from LGC, UK. The amplification parameters on the compatible PCR (GeneAmp 9700) and real-time PCR machines (Roche LightCycler 480 II) were optimised. Microtiter 96- and 384-well plates compatible with the different machines were acquired from Roche, Germany. We also optimised the FLUOstar Omega microplate reader for fluorescence measurement of amplified products following the manufacturer's manual. Then, we ran a KASP trial kit provided freely by LGC Biosearch to test for functionality with the different amplification equipment.

### Application of the optimised genotyping workflow

Following the KASP set-up, we genotyped plant samples for QC and MAS in-house, with low-density markers. The QC genotyping ensured on-time identification of errors and mislabeling in inbred lines and false hybrids in F _1_ maize breeding populations. Using the in-house KASP genotyping platform significantly reduced genotyping cost and time compared to outsourcing.

**Figure 2.**
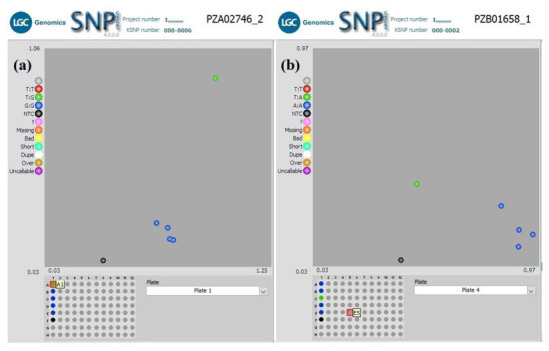
SNPviewer screenshot showing clustering of IITA's maize lines with same lines from three out of four seed sources. ( **a**) Sammaz15-2, -3, and -4 grouped with IITA’s Sammaz15 (blue dots) using SNP PZA02746_2; ( **b**) Sammaz39-2, -3, and -4 grouped with IITA’s Sammaz39 (blue dots) using SNP PZB01658_1. For each SNP marker, blue dots represent homozygous genotypes, green dots represent heterozygote genotypes, and the black dots represent no-template controls (NTC) as indicated on the left side of each image.

**Figure 3.**
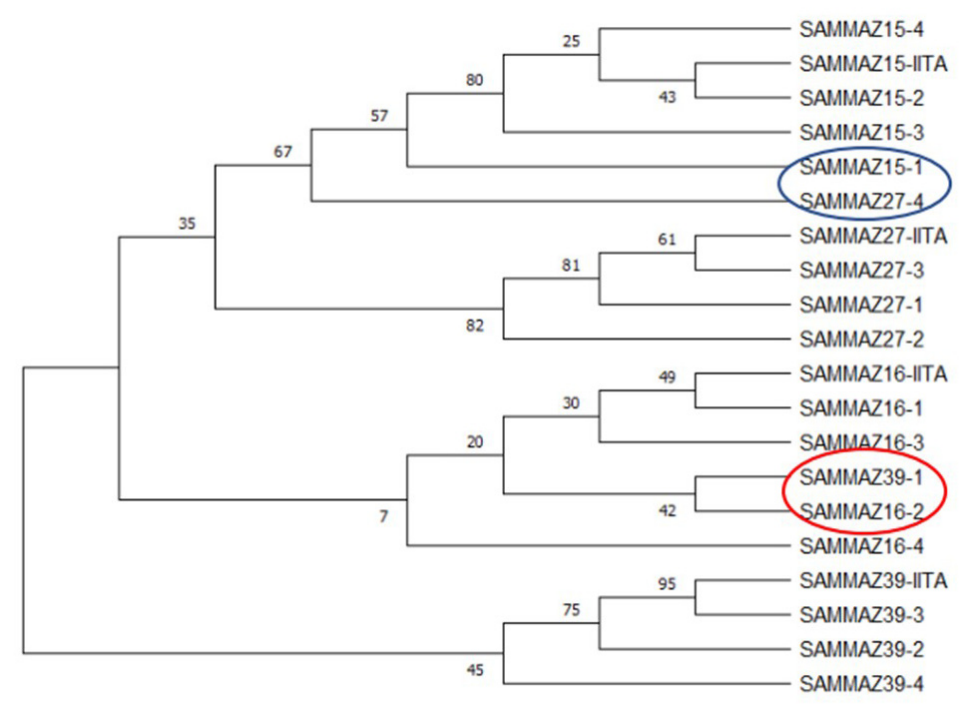
Neighbor-joining tree for four maize varieties taken from five seed sources based on genetic distance, performed with 1,000 bootstrap. Bootstrap values are indicated on the tree branches. The suffixes "-1", "-2", "-3", "-4", and "-IITA", after line name indicate seed source 1, 2, 3, 4, and IITA.

***Genetic identity.*** Using a subset of 28 maize QC KASP SNPs, we were able to identify the genetic origin of a set of twenty well-adapted maize varieties originating from IITA, which were regenerated at four other locations. Genetic identification was performed using the original maize varieties' molecular marker profile and the genetic distance approach. Seed sources having <5% genetic distance were considered the same. The genetic distance among the four original maize lines, and between lines from IITA and each of the four seed sources, was calculated using PowerMaker 3.25 statistical software. The genetic distance among the four designation lines from IITA ranged from 0.0563 to 0.1239, indicating that the lines were different. The genetic distance among the different seed sources of the same line designation was: 0.0105-0.0314 (SAMMAZ15), 0.0105–0.0418 (SAMMAZ16), 0.0105–0.0837 (SAMMAZ27), and 0.000–0.0563 (SAMMAZ39). The SNPviewer, a tool that enables viewing genotyping data as a cluster plot, was used to view and generate an image of the genotyping result. The SNPviewer image showed that designated lines from three out of the four seed sources grouped with lines from IITA ( Figure 2). The dendrogram image ( Figure 3) also showed a grouping of different seed sources of the same line designation except for SAMMAZ39-1, SAMMAZ16-3, and SAMMAZ27-4. This clustering pattern indicates that all seeds from the same line had a common origin. SAMMAZ27-4 appeared to be genetically distant from SAMMAZ27-IITA by 0.0837. However, it grouped with SAMMAZ15 ( Figure 3: blue circle), suggesting a possible mislabeling or mix-up of seeds during harvesting and storage. SAMMAZ16-2 and SAMMAZ39-1 grouped on a different tree limb ( Figure 3: red circle), indicating possible pollen contamination or seed mix-up during handling.

**Figure 4.**
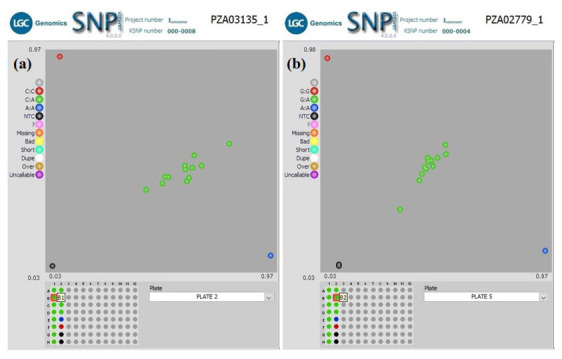
SNPviewer screenshot showing the result of hybrids verification in two sets of F _1_ Plants. ( **a**) Genotyping 12 F1 lines produced from a cross between KS23-5 and IITATZI1653, using SNP PZA03135_1. ( **b**) Genotyping of 12 F1 lines produced from a cross between KS23-5 and IITATZI1667, using SNP PZA02779_1. For each SNP marker, blue dots represent homozygous parental genotype reported by FAM, red dots represent homozygous parental genotype reported by HEX, green dots represent heterozygous hybrid genotypes, and the black dots represent no-template controls (NTC). **Legend**: FAM = Carboxyfluorescein; HEX = Hexachloro-fluorescein.

***Hybrid verification.*** In another QC experiment using our workflow, we screened two groups of F _1_ plants for hybrid verification, including their parental inbred lines, with 10 KASP SNP markers. The parental inbred lines were screened with an initial 50 KASP SNP taken from a defined panel of maize QC KASP markers to identify polymorphic markers. Only 10 KASP markers were polymorphic between the parental lines and were used to screen the F _1_ plants to verify their parentage. Cluster analysis of Group1 F _1_s ( Figure 4) grouped the genotypes into three clusters. The heterozygous F _1_ progenies were in the middle of the plot, and the homozygous parental inbred lines diverged from each other (along the X- and Y-axis of the plot) for all ten markers. This clustering pattern indicates that the F _1_ progenies were true hybrids. Similar clustering was observed among F _1_s in Group 2 except in Set 3b, where 38 F _1_s grouped with parental genotypes. The homozygous F _1_s could be due to contamination from foreign pollens during the crossing in the field or seed mix-up during storage or planting.

***Marker-assisted backcrossing.*** We performed multiple field selections annually by applying our workflow in MAS projects, which accelerated the maize breeding process. For instance, in the MABC project, a set of trait-specific KASP SNPs was used to select 24 BC _1_S _2_ maize lines potentially introgressed with resistance to aflatoxin accumulation after four selection cycles in less than two years. Potentially introgressed lines are now undergoing field evaluation under artificial infestation for resistance to aflatoxin accumulation. The result of the MAS of high PVA lines, on the other hand, identified nine out of 70 inbred maize lines harbouring favourable alleles of the *crtRB1* gene, which is associated with high PVA content in maize.

## Discussion

There are different methods of plant tissue sampling, including collecting samples in silica gel ^34^, NaCl/CTAB ^35^, alcohol ^36^, blotter paper, gel pack, dry ice, and liquid nitrogen ^37^. These methods provide reasonably good quality and quantity of DNA for molecular marker genotyping. However, deciding which method to use is based on the number of samples and distance from the field to the laboratory ^37^. We routinely use wet ice in Styrofoam boxes and cooler bags. It is cost-effective and suitable for close-proximity sample collection, and leaf samples are preserved by freeze-drying ^38^ before DNA extraction. We collected fresh leaf tissues directly into 96-well extraction tubes rather than the traditional jute or tea bags, which means our procedure provides high throughput sampling. This sampling process also ensured that sample DNA was not degraded by prolonged exposure of leaf tissues to moisture as it occurs in post-freeze drying cutting of leaf tissues stored in jute and tea bags.

Our protocol aimed to extract high-quality DNA suitable for KASP genotyping from a smaller amount of leaf tissues. The reduced sample volume lowered the cost of reagents and the time for DNA extraction. The automated grinding in 96-well plates increased throughput and minimised the time required for manual grinding. Thus, this method would benefit MAS breeding programs that often screen thousands of plant samples each season ^39^. A similar high-throughput result was achieved by Anderson *et al.* (2018) ^40^. They optimised the DNA extraction method by Whitlock *et al.* (2008) ^41^, used a 96-well plate for extraction and achieved a consistent yield across the plate with a low failure rate.

Three steps of the original DArT DNA extraction method were slightly modified to achieve our aim. The first modification was made in the sample grinding step, where we used dried leaf tissues instead of fresh ones;—using dried samples enabled high-throughput grinding using a Geno/Grinder, reducing the time used in manual grinding with liquid nitrogen. The second modification was at the alcohol precipitation step: the sample tubes were incubated at -20°C for 30 minutes after adding the ice-cold isopropanol, instead of only mixing by inversion. This incubation is necessary for slow and complete DNA precipitation. The third modification was reconstituting the DNA pellet: we dissolved the DNA in a solution of nuclease-free water and RNaseA instead of using a Tris-EDTA (TE) buffer to prevent the chelating effect of EDTA on Mg ^2+^ during PCR ^42, 43^. The success of the KASP genotyping experiment is dependent on the quality and quantity of genomic DNA. Usually, a final minimum DNA concentration of 5 ng/µl is required for maize, to generate clear and consistent allele calls using the KASP assay ^44^. Our slightly modified DNA extraction method provided good quality DNA, suitable for KASP genotyping. Jain *et al.* (2013) extracted suitable quality DNA from honey that was amplifiable by PCR, using an optimised DArT DNA extraction protocol.

Some commonly used DNA quality and quantity analysis methods include agarose-gel electrophoresis, fluorescence, and Ultraviolet (UV) absorbance-based measurement ^37^. Fluorescence-based measurement using DNA-binding dyes such as PicoGreen is fast, sensitive, and dsDNA-specific; however, it comes with the DNA-binding reagent's added cost ^45, 46^. Agarose gel electrophoresis is laborious and carries the risk of exposure to hazardous chemicals like ethidium bromide ^46^. The UV absorbance measurement is the most common DNA quantitation method. It is based on DNA absorbing UV light at a specific wavelength; DNA concentration is calculated by measuring the absorbance at 260nm and using the relationship A260 of 1.0 equals 50 µg/ml pure dsDNA ^45^. DNA purity is estimated based on two UV absorbance ratios: A260/A280 ≥1.7 and A230/A260 ≥ 1.5 for pure DNA ^45^. Our workflow optimized the nucleic acid quantitation method to a high throughput using a microplate reader and 96- and 384-well plates. The FLUOstar microplate reader uses ultrafast UV/Vis spectrometers for absorbance measurements, measuring 96 samples (96-well plate) to 384 samples (384-well plate) simultaneously within one second per well. It combines speed and the acquisition of complete absorbance spectra (220 to 1000 nm), making it ideal for nucleic acid quantification ^47^.

Although outsourcing KASP offers a lower cost per data point, this lower genotyping cost is usually driven by a high volume of samples, impracticable for most MAS projects genotyping smaller sample volumes with select markers ^48^. Our in-house genotyping system provides reduced cost, mainly from logistics, and faster data turnaround times, ultimately accelerating the genotyping workflow.

A few studies serve as the benchmark for QC analysis in maize using the KASP genotyping system. Semagn *et al.* (2012) suggested using a subset of 50 to 100 KASP markers for routine QC; Chen *et al.* (2016) used a smaller subset of markers (10 markers) to assess mislabeling of entries across a panel of CIMMYT Maize Lines (CMLs) achieving up to 99% detection probability. The latter also proposed using a rapid QC approach, with a smaller subset of markers, to ensure effective QC, lower genotyping costs, and shorten data turnaround time during seed production. Using a subset of markers, we were able to identify seed mix-up and labelling errors. For instance, the grouping of SAMMAZ27-4 with SAMMAZ15 ( Figure 3: blue circle) suggests a possible mislabeling or mix-up of seeds during harvesting and storage. Also, the grouping of SAMMAZ16-2 and SAMMAZ39-1 ( Figure 3: red circle) indicates possible pollen contamination or seed mix-up during handling. Similar errors due to seed mix-up and contamination were reported in Semagn *et al.* (2012), where 50 KASP SNPs were used to determine genetic identity among two to four seed sources of the same inbred line. Ertiro *et al*. (2015) also reported a high discrepancy in genetic purity and identity by the origin of seed sources irrespective of the genotyping platform used. They concluded that using a small subset of pre-selected high-quality markers was sufficient for performing QC analysis using low-marker density genotyping platforms like KASP. This study showed that the rapid QC method using 28 KASP SNPs efficiently distinguished the four maize varieties taken from five seed sources.

Hybrid verification is often performed during seed production or population breeding to confirm that a particular hybrid is derived from the intended parental lines (free from contamination by foreign pollens). Reducing the data turnaround time is essential to ensure that an accurate hybrid is selected to be carried forward in breeding programs or dissemination to farmers in seed production ^32^. A reduced turnaround time also saves the cost of inputs applied to undesired genotypes since they can be discarded as soon as they have been identified upon genotyping. Our expedited workflow was able to achieve this. The possibility of contamination by self-pollination or foreign pollen exists; as such, hybrid verification is necessary to enable a seed producer to check whether accurate crosses are made for the production of the hybrid; this increases the confidence of the end-users on the quality and integrity of seeds produced ^32^. Our results showed that 10 KASP markers were sufficient in distinguishing between maize parental inbred lines and identified true hybrid lines, residual contaminations, and possible sampling errors. A small subset of KASP markers has also been used to verify hybrids in other plant species. Patterson *et al.* (2017) ^49^ achieved a highly accurate picture of *Myriophyllum* species distribution dynamics in North American lakes by genotyping 39 individuals from both parental watermilfoil and their hybrids, using a subset of three KASP markers. Osei *et al.* (2020) ^50^ used 38 KASP markers to screen tomato genotypes to identify true F _1_ hybrids for the possible development of inbreds with long shelf life through marker-assisted backcrossing (MABC).

Following our optimised workflow, we were able to identify high-PVA maize lines harbouring the favourable allele of the *crtRB1* gene, which could serve as donor lines for the maize PVA breeding program. The KASP-based selection of aflatoxin-resistant maize lines promises to fast-track the development of tropical lines resistant to aflatoxin, which will contribute to genetic gain in maize production. Similar success was achieved by the Biotechnology Center of the University of California, Davis, USA, where KASP SNPs associated with *Phytophthoria capsici* resistance were used to identify and selectively breed pepper strains ^51^. So far, we have generated over 2,000 data points using our in-house genotyping workflow. Applying our optimised workflow to the QC and MAS experiments outlined above reduced the volume of reagents and consumables used, shortened the data turnaround, and ultimately accelerated the crop improvement process.

## Conclusions

This study describes for the first time an improvement of an entire conventional DNA-based genotyping workflow, including the benchmark KASP genotyping platform in-house in our facility to fast-track molecular marker-based selection for crop improvement. We acknowledge the initial capital investment to procure some of these instruments. However, it is not always necessary to equip each lab or breeding program. The use of shared facilities locally and regionally helps overcome the high cost of essential instruments. The improved genotyping workflow promises to accelerate the marker-assisted selection process and push crop improvement activities to attain the yield potential over a shorter time period. The result of this work can be readily adopted by national institutions, public and small plant breeding laboratories in developing countries to accelerate molecular marker-based genotyping for crop improvement activities, including QC and MAS. The results will also be helpful to accelerate the QC activities of seed producers and facilitate cultivar identification and adoption-tracking studies.

## Data availability

### Underlying data

Figshare: SNP data for "Developing and deploying an efficient genotyping workflow for accelerating maize improvement in developing countries.", https://doi.org/10.6084/m9.figshare.17157914 ^52^.

This project contains the following underlying data:

- Supplementary Table 1. List of trait-specific KASP SNPs used in the MAS experiment with sequence information
- Supplementary Table 2. List of KASP SNPs used in the QC experiments with sequence information

Data are available under the terms of the Creative Commons Zero "No rights reserved" data waiver (CC0 1.0 Public domain dedication).
