# Supplementary material for: Developing and deploying an efficient genotyping workflow for accelerating maize improvement in developing countries
Source: Gates Open Res. 2022 Aug 3;6:3. Originally published 2022 Jan 17. [Version 3] doi: 10.12688/gatesopenres.13338.3 (PMC10313903; doi:10.12688/gatesopenres.13338.3)
Supplement: Peer review history

# Peer review: Reviewer response for version 3

**DOI:** 10.21956/gatesopenres.15059.r33596

**Published:** 2023-07-13

**Authors:** Ruairidh J H Sawers

**Affiliations:** 1 Plant Science, The Pennsylvania State University - University Park Campus, University Park, PA, USA

A nice survey of the options and applications for genotyping in breeding programs with useful details and experience of establishing an in-house KASP genotyping platform. The manuscript is strong on specific details that will be helpful to other researchers attempting similar work.

 I’ve included a few general comments/thoughts that might be useful to consider.

 Other reviewers have commented on the usefulness of a more complete economic breakdown. I’m not sure specific numbers (such as pricing given in the introduction) are necessarily that useful as they will no-doubt change. However, I would agree that more discussion of the relative costs of given approaches and different scales would be helpful. More generally, although services for the same technology are compared, less attention is given to different approaches. Indeed, given the context of extending access to molecular platforms, if would be informative to say more about the costs of using these technologies at all in comparison to conventional methods. For the specific KASP application, more could be said about the costs of primer design – especially if not using a crop well served with existing sequences – and synthesis.

 The case studies are informative but would benefit from providing more information on the markers (for example, map position) and the sample genotypes. More could be said about the selection of markers, and specifically more discussion of how many markers are actually needed for a given application (based on these empirical examples, as much as prior literature). Results for selected markers are presented visually. Are these “typical” examples? Can more of a summary be given as to how many markers “worked”, and how reproducible and reliable the results were? The results presented clearly separate genotypic classes (except for one highlighted individual). Was this always the case? Was calling of heterozygotes always robust? Was any additional confirmation performed? Would it be possible to estimate the rate of miscalling, either per marker or generally for the platform?   

 The cluster/calling of the KASP signal is most robust when each genotypic class is represented by multiple individuals. In Fig.2 the sample size is small, and the homozygous T/T class is not represented. In isolation such sampling may complicate “calibration” of the heterozygous calls. Similarly, in Fig. 4 only a single sample is typed for each of the two parental homozygotes. The examples presented look nice and clear, but was this always the case for all markers typed on these samples?

 It wasn’t entirely clear what was done in the dendrogram in Fig. 3. How many markers were used? How was this number/set determined? Were they spread throughout the genome? Do these lines show a level of heterozygosity? As above, was there any ambiguity/error in calling?

 It’s a small detail, but at times the use of the term “line” was a little confusing. It can help to keep “line” to refer to inbred (highly homozygous) stocks. While an F1 is typically a cross between two lines, don’t refer the F1 as an “F1 line” etc. The expectation with regard to heterozygosity – and the requirement to accurately make het calls – is directly relevant to selection and use of a genotyping platform.

Is the rationale for developing the new method (or application) clearly explained?

Yes

Is the description of the method technically sound?

Yes

Are the conclusions about the method and its performance adequately supported by the findings presented in the article?

Partly

If any results are presented, are all the source data underlying the results available to ensure full reproducibility?

Partly

Are sufficient details provided to allow replication of the method development and its use by others?

Partly

Reviewer Expertise:

Maize diversity, genetics and genomics; crop nutrition; mycorrhizal symbiosis

I confirm that I have read this submission and believe that I have an appropriate level of expertise to confirm that it is of an acceptable scientific standard.

# Peer review: Reviewer response for version 3

**DOI:** 10.21956/gatesopenres.15059.r33600

**Published:** 2023-06-30

**Authors:** Karthikeyan Adhimoolam

**Affiliations:** 1 Horticulture, Jeju National University, Jeju-si, Jeju-do, South Korea; 2 Tamil Nadu Agricultural University, Madurai, Tamil Nadu, India

This study demonstrates the improved genotyping workflow for maize improvement in developing countries. I recommend the manuscript for indexing.

 However, I recommend drawing a better workflow figure instead of Figure 1. And present some data (i.e., Table) to support the cost-effectiveness. Also, I suggest the authors improve the language.

Is the rationale for developing the new method (or application) clearly explained?

Yes

Is the description of the method technically sound?

Yes

Are the conclusions about the method and its performance adequately supported by the findings presented in the article?

Yes

If any results are presented, are all the source data underlying the results available to ensure full reproducibility?

Yes

Are sufficient details provided to allow replication of the method development and its use by others?

Yes

Reviewer Expertise:

Plant molecular breeding and plant genomics

I confirm that I have read this submission and believe that I have an appropriate level of expertise to confirm that it is of an acceptable scientific standard.

# Peer review: Reviewer response for version 3

**DOI:** 10.21956/gatesopenres.15059.r32358

**Published:** 2022-09-16

**Authors:** Bhoja R. Basnet

**Affiliations:** 1 International Maize and Wheat Improvement Center (CIMMYT), Texcoco, Mexico

Dear authors, thank you for sincerely handling the reviews and putting your best effort into addressing those comments and concerns. I do not have further queries. I believe we let readers, the fellow researchers, and the broader scientific community judge the merit of this research.

Is the rationale for developing the new method (or application) clearly explained?

Partly

Is the description of the method technically sound?

Yes

Are the conclusions about the method and its performance adequately supported by the findings presented in the article?

Yes

If any results are presented, are all the source data underlying the results available to ensure full reproducibility?

Partly

Are sufficient details provided to allow replication of the method development and its use by others?

Partly

Reviewer Expertise:

Plant breeding and genetics, genomics, quantitative genetics, and breeding program optimization.

I confirm that I have read this submission and believe that I have an appropriate level of expertise to confirm that it is of an acceptable scientific standard.

# Peer review: Reviewer response for version 2

**DOI:** 10.21956/gatesopenres.14950.r32187

**Published:** 2022-07-08

**Authors:** Bhoja R. Basnet

**Affiliations:** 1 International Maize and Wheat Improvement Center (CIMMYT), Texcoco, Mexico

It should go through one more round of revision. Please address the following concerns:

1. Introduction P3L3: "However, breeders often want to fingerprint a few dozen lines urgently for identity or parentage analysis for example". Please rephrase this sentence.
2. Please provide well-articulated one or two objectives of this research (towards the end of the Introduction section).
3. Introduction section P6L1: "Here we utilized the KASP assay" - revise as "In this study, we utilized...".
  Introduction P6L6: reference 27 does not provide any account of soybean - the paper is about maize. Please verify this information and correct it as needed.
4. Methods P1L3: "Well-adopted" should be changed to "well-adapted".
5. Method P1 Last sentence: The plants were grown, not raised.
6. Table 1: This is a piece of good information. However, I ask you to provide the exact number of genotypes and the samples within each genotype for all the groups (please add additional columns as needed). 
7. Use of BC1S2 does not seem to be reliable in this study unless you verify the selection with phenotypic data to estimate the sensitivity and specificity of the marker assessment. However, it doesn't seem to harm the manuscript either.
8. One important analysis I would like to suggest to add to this study is HYBRID VERIFICATION. Please prepare a data table for each sample - identified within each genotype (F1), such as order the column as F1 cross name / no, sample #, Marker gen _P1, Marker gen_P2, Observed F1 gen, True Hyb (Yes or no), if not if the F1 gen is observed as maternal or paternal type, etc. Then please assess the true to hybrid types or % hybridity within each genotype (using samples within cross) and across all samples. Then also revise your results section with a detailed discussion on how this assay is helpful to discriminate true-to-type hybrids and also describe potential bias caused by the assay itself - genotyping error or so using data on samples within each genotype. 
9. Did you sample multiple samples within each plant? If so, please revise the results section accordingly. 

Is the rationale for developing the new method (or application) clearly explained?

Partly

Is the description of the method technically sound?

Yes

Are the conclusions about the method and its performance adequately supported by the findings presented in the article?

Yes

If any results are presented, are all the source data underlying the results available to ensure full reproducibility?

Partly

Are sufficient details provided to allow replication of the method development and its use by others?

Partly

Reviewer Expertise:

Plant breeding and genetics, genomics, quantitative genetics, and breeding program optimization.

I confirm that I have read this submission and believe that I have an appropriate level of expertise to confirm that it is of an acceptable scientific standard, however I have significant reservations, as outlined above.

# Response

**Published:** 2022-07-28

**Authors:** Queen Offornedo

**Affiliations:** International Institute of Tropical Agriculture, Nigeria

The authors thank the reviewers for taking the time to review the manuscript and raise critical issues. We trust that addressing these issues will immensely improve the paper. Below are the responses to the concerns raised.

**Reviewer 2 comment**: Introduction P3L3: "However, breeders often want to fingerprint a few dozen lines urgently for identity or parentage analysis, for example". Please rephrase this sentence.

**Author's response**: The sentence has been rephrased: "Even though large volume sizes can be consolidated and shipped for genotyping, there are times when breeders and partners may want to fingerprint a few dozen lines for identity or parentage analysis for quick decision making."

**Reviewer 2 comment**: Please provide well-articulated one or two objectives of this research (towards the end of the Introduction section).

**Author's** **response**: The objective of the research has been rephrased "This study aims to develop a genotyping workflow optimized for cost-effective and fast turn-around time that can be deployed by less sophisticated and reasonably equipped laboratories in developing countries, to accelerate maize improvement research."

**Reviewer 2 comment**: Introduction section P6L1: "Here we utilized the KASP assay" - revise as "In this study, we utilized...".

 Introduction P6L6: reference 27 does not provide any account of soybean - the paper is about maize. Please verify this information and correct it as needed.

**Author's response**: The phrase has been corrected.

 The misplaced reference has been replaced. A more suitable reference has been attached to the statement. " Shi, Z., Liu, S., Noe, J. *et al.* SNP identification and marker assay development for high-throughput selection of soybean cyst nematode resistance. *BMC Genomics* 16, 314 (2015). https://doi.org/10.1186/s12864-015-1531-3 **"**

**Reviewer 2 comment**: Methods P1L3: "Well-adopted" should be changed to "well-adapted".

**Authors' response**: The phrase has been modified accordingly.

**Reviewer 2 comment**: Method P1 Last sentence: The plants were grown, not raised.

**Author's response**: The sentence has been modified as requested.

**Reviewer 2** **comment**: Table 1: This is a piece of good information. However, I ask you to provide the exact number of genotypes and the samples within each genotype for all the groups (please add additional columns as needed).

**Author's response**: Table 1 has been modified to accommodate the required information.

**Reviewer 2** **comment**: Use of BC1S2 does not seem to be reliable in this study unless you verify the selection with phenotypic data to estimate the sensitivity and specificity of the marker assessment. However, it doesn't seem to harm the manuscript either.

**Author's response**: The project is still ongoing. Phenotyping at different locations is currently underway. Definitely, we will utilize the phenotype data to verify the markers accuracy.

**Reviewer 2** **comment**: One important analysis I would like to suggest to add to this study is HYBRID VERIFICATION. Please prepare a data table for each sample - identified within each genotype (F1), such as order the column as F1 cross name / no, sample #, Marker gen _P1, Marker gen_P2, Observed F1 gen, True Hyb (Yes or no), if not if the F1 gen is observed as maternal or paternal type, etc. Then please assess the true to hybrid types or % hybridity within each genotype (using samples within cross) and across all samples. Then also revise your results section with a detailed discussion on how this assay is helpful to discriminate true-to-type hybrids and also describe potential bias caused by the assay itself - genotyping error or so using data on samples within each genotype. 

**Author's response**: The genotyping analysis for the hybrid verification experiment is presented in Figure 5 and Table 5, under the Result section. The result section has also been furnished with a detailed discussion on using the KASP assay for hybrid verification and the potential drawback of the technology, as shown below:

***Hybrid verification.*** In another QC experiment using our workflow, we screened two groups of F _1_ plants for hybrid verification, including their parental inbred lines, with 10 KASP SNP markers. The parental inbred lines were screened with an initial 50 KASP SNP taken from a defined panel of maize QC KASP markers to identify polymorphic markers. Only 10 KASP markers, polymorphic between the parental lines, were used to screen the F _1_ plants to verify their parentage. The KASP genotyping assay was useful in distinguishing between the parental genotypes and identifying the true hybrid lines. Cluster analysis of Group1 F _1_s ( Figure 4) grouped the genotypes into three clusters. The heterozygous F _1_ progenies were in the middle of the plot, and the homozygous parental inbred lines diverged from each other (along the X- and Y-axis of the plot) for all markers. The genotyping result (Table 5) and the clustering pattern indicate that the F _1_ progenies were true hybrids. Similar clustering was observed among F _1_s in Group 2 except in Set 3b, where 38 F _1_s were grouped with parental genotypes. The homozygous F _1_s could be due to contamination from foreign pollens during the crossing in the field or seed mix-up during storage or planting.

 Nonetheless, the KASP genotyping assay suffers some genotyping errors, especially during the automatic calling of genotypes. For instance, one F1 line (SCH-4) developed from the bi-parental cross, KS23-6 and IITATZI1653, appeared to cluster with the parent 2 (IITATZI1653) when genotyped with marker PZB01658_1 (Figure 5). The datapoint representing IITATZI1653 (Figure 5, information in the yellow square) was plotted higher up, away from the X-axis, which brought it closer to the datapoint representing SCH-4 plotted slightly away from the other F1s in the middle. Because genotype calls are generated based on the relative position of datapoints on the plot, SCH-4 was automatically called as the nearby parental genotype, A:A, which was an error seeing that line SCH-4 was heterozygous (true hybrid) for the rest of the markers. The upward positioning of line IITATZI1653 away from the X-axis could be possibly due to trace contamination of line IITATZI1653 sample DNA with line  KS23-6 sample DNA during sample preparation.  A monomorphic marker is seen in the genotyping of F1 lines developed from the bi-parental crosses KS23-3 x IITATZI1653 using marker PHM5502_31.

**Reviewer 2** **comment**: Did you sample multiple samples within each plant? If so, please revise the results section accordingly.

**Author's response**: I am hoping that I got your question correct here. If you are referring to whether or not we sampled by bulking, the answer is no, except for the MAS experiment for selecting PVA enriched lines, where we bulked ten leaf tissues from 10 plant stands per row.

# Response

**Published:** 2022-08-25

**Authors:** Bhoja R. Basnet

**Affiliations:** CIMMYT, Mexico

1. Before making the final decision, I am still unsure how you controlled or separated genetic purity and genotyping error in the assay. The markers seem to predict the hybrids almost perfectly, with few exceptions. How was that possible? I am not trying to deny the fact, but being curious as it was not the case in wheat.

 2.  My last question was about 'analyzing multiple samples from the same plant - without bulking.' Normal practice in QC for genetic purity and true-to-type hybrid verification is that multiple F1s samples are used (you have done it), and multiple samples within each plant are also used to control the genotyping or other handling errors that may arise during the genotyping workflow. It also gives confidence about the reproducibility of the same results for the same genetic materials.

# Response

**Published:** 2022-09-06

**Authors:** Queen Offornedo

**Affiliations:** International Institute of Tropical Agriculture, Nigeria

**Reviewer 2 comment: **Before making the final decision, I am still unsure how you controlled or separated genetic purity and genotyping error in the assay. The markers seem to predict the hybrids almost perfectly, with few exceptions. How was that possible? I am not trying to deny the fact, but being curious as it was not the case in wheat.

**Author's response**: We are unsure what the case is when genotyping wheat using the KASP assay. However, the prediction of the maize hybrids could have been aided by the fact that maize is diploid, unlike wheat which is polyploid. The polyploid nature of wheat could make it challenging to distinguish between heterozygous and homozygous hybrid lines using KASP assays. Also, our genotyping is complemented by careful crossing and sample collection in the field and meticulous handling of the samples. The KASP assay genotyping prep is handled by select individuals and carried out in a dimmed light PCR workstation to minimize contamination and avoid activating the light-sensitive fluorophores in the KASP Mastermix. Furthermore, we first selected polymorphic markers that distinguished the parents, and only those were used to identify the hybrid lines. As such, any given F1 line is either a hybrid or not. Given the robustness and accuracy of the KASP assay, the use of multiple markers, and careful handling of the sample analysis, the chance of error is minimal.

**Reviewer 2 comment:**  My last question was about 'analyzing multiple samples from the same plant - without bulking. 'Normal practice in QC for genetic purity and true-to-type hybrid verification is that multiple F1s samples are used (you have done it), and multiple samples within each plant are also used to control the genotyping or other handling errors that may arise during the genotyping workflow. It also gives confidence about the reproducibility of the same results for the same genetic materials."

**Author's Response**: Thank you for clarifying your question.

 For this experiment, 12 different F1 plants per cross were sampled, although the number of samples per plant can vary depending on the breeder's request based on the number of seeds required for the subsequent experiment. We, however, did not do a duplicate analysis of the F1 plants. We acknowledge the importance of having technical replicates in an experiment; however, the accuracy of the KASP assay is well established (as cited in our manuscript). The specificity of the KASP assay means that even one validated marker can accurately distinguish between parents and offspring, and using up to 10 markers reduces the chance of genotyping error significantly. Therefore, using technical replicates may not be worth the cost; instead, we could increase the number of markers in case of any doubt or possible errors.

# Peer review: Reviewer response for version 2

**DOI:** 10.21956/gatesopenres.14950.r32149

**Published:** 2022-06-20

**Authors:** John Damien Platten

**Affiliations:** 1 International Rice Research Institute, Los Baños, Philippines

I thank the authors for taking the time to address the issues raised in the previous review. I do think there are still some outstanding issues that would substantially improve the case for the work presented:

- Articulation of what use cases are in mind needs to be better. Currently, the articulation is basically "low-throughput applications that may require fast turnaround time". This is true to a certain extent, but not the strongest case; it could easily be argued that any "low-throughput" request could either pay the extra up-front fees, as this will be cheaper than maintaining a lab just for this purpose, or samples aggregated with other larger jobs. In the end, this is essentially saying that because certain breeders were not organised in their workflow, we need to maintain an entire lab for them.
- I still don't see any costing of the procedures. This is sorely needed, even if this costing only includes consumables and not salaries of dedicated staff. It should be compared with service providers for jobs of the same # samples and # SNPs.

Is the rationale for developing the new method (or application) clearly explained?

Partly

Is the description of the method technically sound?

Partly

Are the conclusions about the method and its performance adequately supported by the findings presented in the article?

No

If any results are presented, are all the source data underlying the results available to ensure full reproducibility?

No source data required

Are sufficient details provided to allow replication of the method development and its use by others?

Partly

Reviewer Expertise:

Marker design, marker evaluation, marker QC metrics, marker-assisted introgression, molecular breeding

I confirm that I have read this submission and believe that I have an appropriate level of expertise to confirm that it is of an acceptable scientific standard, however I have significant reservations, as outlined above.

# Response

**Published:** 2022-07-28

**Authors:** Queen Offornedo

**Affiliations:** International Institute of Tropical Agriculture, Nigeria

The authors thank the reviewers for taking the time to review the manuscript and raise critical issues. We trust that addressing these issues will immensely improve the paper. Below are the responses to the concerns raised.

**Reviewer 1 comment**: I thank the authors for taking the time to address the issues raised in the previous review. I do think there are still some outstanding issues that would substantially improve the case for the work presented:

 Articulation of what use cases are in mind needs to be better. Currently, the articulation is basically "low-throughput applications that may require fast turn-around time". This is true to a certain extent, but not the strongest case; it could easily be argued that any "low-throughput" request could either pay the extra up-front fees, as this will be cheaper than maintaining a lab just for this purpose, or samples aggregated with other larger jobs. In the end, this is essentially saying that because certain breeders were not organized in their workflow, we need to maintain an entire lab for them.

**Authors' response**: Besides the fact that the logistics for sending a low sample volume is not cost-effective, we also indicated in our previous response that there is a benefit of turn-around time when we genotype in-house. We had also indicated that neither the lab nor the equipment is procured solely for this genotyping purpose. We reiterate that the lab is a shared facility serving multiple activities, as are the equipment; We have re-purposed the real-time PCR for KASP assay. There is no maintenance cost for this workflow. The only thing dedicated to the workflow is the Klustercaller software.

 We still outsource samples for the routine forward breeding application. However, there are applications where a quick genotyping of a small number of samples has to be done, for QC purposes, for breeders and partner seed companies. In summation, this study is focused on the scientific rigour rather than the business proposition of the technological workflow.

**Reviewer 1 comment**: I still don't see any costing of the procedures. This is sorely needed, even if this costing only includes consumables and not salaries of dedicated staff. It should be compared with service providers for jobs of the same # samples and # SNPs.

**Authors' response**: A statement detailing the cost comparison of the procedure has been included in the Introduction section (P3L6): "The standard cost for routine KASP genotyping by Intertek is $2.6 per sample per 10 SNPs, excluding shipping costs, compared to our in-house genotyping at $2.95."

# Peer review: Reviewer response for version 1

**DOI:** 10.21956/gatesopenres.14581.r31823

**Published:** 2022-03-18

**Authors:** John Damien Platten

**Affiliations:** 1 International Rice Research Institute, Los Baños, Philippines

**Is the rationale for developing the new method (or application) clearly explained?**

 The value proposition for developing the ‘method’ is not well articulated. The authors mention several times the advantages of an in-house genotyping platform, and the general thrust of the paper is describing successful proof-of-concept application of some standard components of a SNP genotyping protocol.  However it is not especially clear if the authors are aiming to establish this service as a cost-effective alternative to outsourcing, to meet a specific need that outsourcing does not meet, or something else.

 Alongside this, it is not clear what the novelty of the new method is. The entire workflow represents an implementation of standard technologies (CTAB extraction, DNA quantification, KASP genotyping). None of these are new techniques, nor is their combination into a genotyping workflow.

**Is the description of the method technically sound?**

 As with point number 1, the overall description is technically sound, but several key details are overlooked. The machinery used in the critical step of plate scanning (actual data acquisition) is described, but key parameters are missing (please substitute equivalent parameters depending on the model of machine):

- What settings are used for lamp energy?
- What filters are used for excitation and emission spectra? This should include part numbers, and technical details of their performance.
- How is the analysis (clustering) done?
- How are results aggregated and conclusions drawn?

**Are sufficient details provided to allow replication of the method development and its use by others?**

 See the previous comments.  Some aspects are adequately described, but some others are sparse on critical technical details.

**If any results are presented, are all the source data underlying the results available to ensure full reproducibility?**

 Largely not applicable.

**Are the conclusions about the method and its performance adequately supported by the findings presented in the article?**

 This does not seem to be the case. In particular, benchmarking data on the capacity, technical performance, cost etc. are lacking. This makes it impossible to judge the merits of this in-house system compared to outsourcing options.

**Overall conclusion:**

 The current manuscript shows ability to technically execute on a relatively small number of samples in a modest timeframe. However to be a substantial contribution in this space, more thought needs to be given to better articulate both the value proposition of the work, and provide some benchmarking data to back this up. For example if the overall purpose is to show the benefit of having an in-house genotyping platform as opposed to (or in addition to) outsourcing options, the following factors and results might be considered:

- What is the value of an in-house system? Turnaround time and flexibility are mentioned, which I agree with. However why is this particularly important, to justify the expense of setting up, maintaining and operating an in-house system? Are there logistical considerations that prevent the use of outsourced options? Is the in-house system functionally *superior* to outsourced options? Is there a particular part of the breeding process that does not lend itself to standard outsourced options – and if so, under what circumstances would it be advisable to use the in-house or outsourced options? See below comments on benchmarking.
- **Full** cost assessment of the in-house system, including salaries of technical staff, machine maintenance and depreciation. Some description of the staff involved (number of positions executing on various duties) would also be helpful.
- Also an assessment of technology life-cycles; genotyping platforms are evolving rapidly. I have seen many cases of expensive machines being purchased, only to sit idle as the technology has moved on even before they are delivered.  KASP is likely to be replaced in the next 5 years. How would the cost of staying up to date and current be factored in?
- Exploration of capacity. The authors mention completing 3 jobs (637 samples) in two weeks. This is plausible based on personal experience, though I have seen in-house systems with far higher throughput. However this is a far cry from handling 20,000 samples at peak operating times. This relates back to the first point.
- Also related to capacity, an exploration of current/anticipated peak demand for the system.
- Technical performance metrics:
  - Average DNA quality.
  - Call rate: what percentage of samples×markers (datapoints) do not amplify?
  - Clarity: What proportion of datapoints are unscorable?
  - Reproducibility: Amongst technical and biological replicates, what proportion of datapoints are scored incorrectly/inconsistently?
- Benchmarking against available outsourcing options. A comparison of parameters such as these:
  - Turnaround time.
  - **Full** cost per sample and per datapoint.
  - Monthly capacity (samples and datapoints).

In-house genotyping platforms can and do have merit and justification. However until these issues can be addressed, the manuscript in its current form offers no fundamental insights into how such a platform could add value to breeding over outsourcing options.

 If the authors can better explain why their hub is superior over other options, backed up with benchmarking data such as specified, this would greatly enhance its value.

Is the rationale for developing the new method (or application) clearly explained?

Partly

Is the description of the method technically sound?

Partly

Are the conclusions about the method and its performance adequately supported by the findings presented in the article?

No

If any results are presented, are all the source data underlying the results available to ensure full reproducibility?

No source data required

Are sufficient details provided to allow replication of the method development and its use by others?

Partly

Reviewer Expertise:

Marker design, marker evaluation, marker QC metrics, marker-assisted introgression, molecular breeding

I confirm that I have read this submission and believe that I have an appropriate level of expertise to state that I do not consider it to be of an acceptable scientific standard, for reasons outlined above.

# Response

**Published:** 2022-03-23

**Authors:** Queen Offornedo

**Affiliations:** International Institute of Tropical Agriculture, Nigeria

The authors are very grateful to the reviewer who thoroughly read the manuscript and raised critical issues. We believe that addressing these issues will immensely improve the paper. Below are the responses to the issues raised. We noticed that our core message, which is complementing outsourcing in some situations, was not clearly articulated. We await additional reviewers' comments. We plan to revise the manuscript based on our response below and additional reviewers' comments.

**Is the rationale for developing the new method (or application) clearly explained?**

**Reviewer's comment**:

 The value proposition for developing the 'method' is not well articulated. The authors mention several times the advantages of an in-house genotyping platform, and the general thrust of the paper is describing successful proof-of-concept application of some standard components of a SNP genotyping protocol. However it is not especially clear if the authors are aiming to establish this service as a cost-effective alternative to outsourcing, to meet a specific need that outsourcing does not meet, or something else.

**Author's response:** 

 The core message of our paper is to complement, not establish an alternative, to outsourcing (please see the third paragraph on page 3). We duly recognize the cost-effectiveness of the genotyping platform facilitated by CGIAR platforms such as HTPG/EiB. The need for developing such in-house workflow had been prompted by the following factors:

1. The current available genotyping platforms have a minimum sample size requirement. For instance, the EiB facilitated genotyping at Intertek costs $2 / sample if the user orders genotyping of 1536 samples (a set of 16 plates; four plates are acceptable but price increases). Breeders often want to fingerprint a few dozen lines urgently for identity or parentage analysis. In such cases, sending less than the minimum number of samples is not only more priced per datapoint but entails shipping cost and a turn-around time of 2-3 weeks. Using other markers, such as SSR, is more expensive and cumbersome. The use of genotyping systems such as KASP alleviates all these issues.
2. Logistical issues related to shipping by courier: In this part of the world, courier services are not very satisfactory and reliable, often resulting in damage to samples in transit or longer than normal delays, which may reduce the quality of perishable specimens. If a reasonably affordable system is available locally, it can circumvent such problems.
3. The instruments used for this work are all standard instruments available in most molecular biology labs. Our workflow shows the re-purposing of these instruments for the genotyping workflow. For instance, the qPCR machine, which is mostly used for expression analysis, was adapted to KASP genotyping with the installation of appropriate software for SNP calling. Likewise, the Fluostar plate reader was used for plate-level DNA quantification in lieu of single sample analysis by Spectrophotometer.

**Reviewer's comment**:

 Alongside this, it is not clear what the novelty of the new method is. The entire workflow represents an implementation of standard technologies (CTAB extraction, DNA quantification, KASP genotyping). None of these are new techniques, nor is their combination into a genotyping workflow.

**Author's response:**

 This manuscript is about a workflow that combines carefully chosen and optimized best practices in lab techniques at different stages of genotyping to address pertinent problems faced by researchers in Sub-Saharan Africa (SSA). For users who want to genotype few samples quickly, some bottlenecks in the workflow has to be removed. Currently, the DNA extraction throughput has improved by isolating and quantifying DNA at a plate level (i.e., processing 96 samples simultaneously). Secondly, genotyping by other systems such as SSR is not cost-effective. Therefore, by implementing such a workflow, we could generate quality data quickly for application in the breeding pipeline. It should be noted that not many labs in developing countries are capable of using the KASP system in-house.

**Is the description of the method technically sound?**

**Reviewer's comment**:

 As with point number 1, the overall description is technically sound, but several key details are overlooked. The machinery used in the critical step of plate scanning (actual data acquisition) is described, but key parameters are missing (please substitute equivalent parameters depending on the model of machine):

- What settings are used for lamp energy?
- What filters are used for excitation and emission spectra? This should include part numbers,and technical details of their performance.
- How is the analysis (clustering) done?

How are results aggregated and conclusions drawn?

**Author's response:**

 The required information will be incorporated in the revised manuscript under the subsection "KASP genotyping and data analysis", as explained below:

 The description of the parameters for the LC480 II qPCR machine is outlined in the LC480 manual. To perform the KASP genotyping experiment on the LC480 II machine, we used the Endpoint Genotyping Analysis module within the LightCycler software, adjusting the parameters as outlined in the KASP genotyping protocol provided by LGC Biosearch Technologies. The Endpoint genotyping analysis module is based on the use of dual hydrolysis probes, which are designed for wild-type and mutant target DNA and are labelled with different dyes (FAM and HEX). However, when using a non-qPCR machine (such as the GeneAmp PCR System 9700) for amplification, a third colour probe (ROX) normalizes the fluorescence measurement. The LightCycler software within the LC480 II machine determines the sample genotypes automatically by measuring the intensity distribution of the two probes after a PCR amplification step. The relative dye intensities are then visualized in a scatter (cluster) plot that discriminates them as wild-type, heterozygous mutant, or homozygous mutant samples. The LightCycler software automatically groups similar samples and assigns genotypes based on the intensity distribution of the two dyes. The KASP amplification conditions included one cycle of KASP unique Taq activation at 94°C for 15 min, followed by 36 cycles of denaturation at 94°C for 20 s, and annealing and elongation at 60°C (dropping 0.6°C per cycle) for 1 min. Endpoint detection of the fluorescence signal was acquired for 1 min at 30°C when using the LightCycler 480 II real time-PCR System or read using the FLUOstar Omega Microplate reader (BMG Labtech, SA) when using the GeneAmp PCR System 9700. For fluorescence detection, the filter combination for the Excitation and Emission wavelength of both dyes was set at 465 – 533 (FAM) and 523 – 568 (HEX), respectively, when using LC480 II, and 485 - 520 (FAM), 544 - 590 (HEX) and 584 - 620 (ROX) when using FLUOstar Omega Microplate reader. The genotype calls were exported from the LightCycler software as fluorescent intensities of each sample in ".txt" file format and imported for analysis in the KlusterCaller analysis software (LGC Biosearch Technologies). The KlusterCaller software adjusted the cluster plot axes to enable the proper calling of genotypes. The genotype calls were grouped as homozygous for allele X (allele reported by FAM, X-axis), homozygous for allele Y (allele reported by HEX, Y-axis), heterozygous (alleles reported by FAM and HEX, between X- and Y-axis), or uncallable. The result from the KlusterCaller was exported in two file formats (".csv" and ".txt"). The ".csv" file was imported into the SNPviewer2 version 4.0.0 software (LGC Biosearch Technologies), where the cluster plot image was viewed and downloaded for publication. The genotype calls in the ".txt" file were used to calculate the genetic distance using the PowerMaker  3.25 statistical software.

**Are sufficient details provided to allow replication of the method development and its use by others?**

**Reviewer's comment:**

 See the previous comments. Some aspects are adequately described, but some others are sparse on critical technical details.

**If any results are presented, are all the source data underlying the results available to ensure full reproducibility?**

**Reviewer's comment:**

 Largely not applicable.

**Are the conclusions about the method and its performance adequately supported by the findings presented in the article?**

**Reviewer's comment:**

 This does not seem to be the case. In particular, benchmarking data on the capacity, technical performance, cost etc. are lacking. This makes it impossible to judge the merits of this in-house system compared to outsourcing options.

**Author's response:**

 As mentioned above, the aim of the publication is not to replace outsourcing and we do not envisage competition with outsourcing. We are fully aware of the cost-effectiveness of the highly automated/robotic genotyping services accessible to us. We frequently use these services. The rationale for the workflow is to process fewer samples quickly. The time saved is invaluable. Loss of samples during shipments is a setback, which cannot be monetized easily.

**Overall conclusion:**

**Reviewer's comment:**

 The current manuscript shows ability to technically execute on a relatively small number of samples in a modest timeframe. However to be a substantial contribution in this space, more thought needs to be given to better articulate both the value proposition of the work, and provide some benchmarking data to back this up. For example if the overall purpose is to show the benefit of having an in-house genotyping platform as opposed to (or in addition to) outsourcing options, the following factors and results might be considered:

**Author's response:**

 We do not intend to establish a rival genotyping service to replace outsourcing. As explained above, this workflow is what we are using for a while now for processing a smaller number of samples (smaller than the minimum sample required by service vendors). This gives the flexibility to assay multiple crops with multiple markers in a single plate in a matter of hours. We are a small group dedicated to maize genomics. All we want is to share our methods with partners in the same situation. As can be seen from the stats of the preprint, our manuscript is already making an impact with 20 downloads and 150 views.

**Reviewer's comment:**

 What is the value of an in-house system? Turn-around time and flexibility are mentioned, which I agree with. However why is this particularly important, to justify the expense of setting up, maintaining and operating an in-house system? Are there logistical considerations that prevent the use of outsourced options? Is the in-house system functionally superior to outsourced options? Is there a particular part of the breeding process that does not lend itself to standard outsourced options – and if so, under what circumstances would it be advisable to use the in-house or outsourced options? See below comments on benchmarking.

**Author's response:**

 Addressed above and below.

**Reviewer's comment:**

 Full cost assessment of the in-house system, including salaries of technical staff, machine maintenance and depreciation. Some description of the staff involved (number of positions executing on various duties) would also be helpful.

**Author's response:**

 Not applicable.

**Reviewer's comment:**

 Also an assessment of technology life-cycles; genotyping platforms are evolving rapidly. I have seen many cases of expensive machines being purchased, only to sit idle as the technology has moved on even before they are delivered. KASP is likely to be replaced in the next 5 years. How would the cost of staying up to date and current be factored in?

**Author's response:**

 We agree that the genotyping platforms are evolving rapidly. To make it clear, we have not purchased instruments solely for this technique. We have only re-purposed the existing machines. Both the qPCR machine and the plate reader have high demands for other uses. If we cease to use these machines for the KASP system, the normal utilization of the machines will continue. 

**Reviewer's comment:**

 Exploration of capacity. The authors mention completing 3 jobs (637 samples) in two weeks. This is plausible based on personal experience, though I have seen in-house systems with far higher throughput. However this is a far cry from handling 20,000 samples at peak operating times. This relates back to the first point.

**Author's response:**

 Our response here is related to the above explanation. When we have a large volume of samples, we use low-density and mid-density genotyping service providers.

**Reviewer's comment:**

 Also related to capacity, an exploration of current/anticipated peak demand for the system.

**Technical performance metrics:**

- Average DNA quality.
- Call rate: what percentage of samples × markers (datapoints) do not amplify?
- Clarity: What proportion of datapoints are unscorable?
- Reproducibility: Amongst technical and biological replicates, what proportion of datapoints are scored incorrectly/inconsistently?
- Benchmarking against available outsourcing options. A comparison of parameters such as
- these:
- Turn-around time.
- Full cost per sample and per datapoint.
- Monthly capacity (samples and datapoints).
- In-house genotyping platforms can and do have merit and justification. However until these issues can be addressed, the manuscript in its current form offers no fundamental insights into how such a platform could add value to breeding over outsourcing options.

If the authors can better explain why their hub is superior over other options, backed up with benchmarking data such as specified, this would greatly enhance its value.

**Author's response:**

 As mentioned above, the aim of the publication is not to replace or compete with outsourcing, rather complement it, particularly in cases of a small volume of samples that are not cost-effective for outsourcing.

**Is the rationale for developing the new method (or application) clearly explained?**

**Reviewer's comment: **Partly

**Is the description of the method technically sound?**

**Reviewer's comment: **Partly

**Are sufficient details provided to allow replication of the method development and its use**

**by others?**

**Reviewer's comment: **Partly

**If any results are presented, are all the source data underlying the results available to ensure full reproducibility?**

**Reviewer's comment: **No source data required

**Are the conclusions about the method and its performance adequately supported by the findings presented in the article?**

**Reviewer's comment: **No

**Competing Interests:**

 No competing interests were disclosed.
